# Nematicidal Activity of Volatiles against the Rice Root-Knot Nematode and Environmental Safety in Comparison to Traditional Nematicides

**DOI:** 10.3390/plants13152046

**Published:** 2024-07-25

**Authors:** Jorge M. S. Faria, Leidy Rusinque, Maria L. Inácio

**Affiliations:** 1 INIAV, I.P., National Institute for Agrarian and Veterinary Research, Quinta do Marquês, 2780-159 Oeiras, Portugal; leidy.rusinque@iniav.pt (L.R.); lurdes.inacio@iniav.pt (M.L.I.); 2 GREEN-IT Bioresources for Sustainability, Instituto de Tecnologia Química e Biológica, Universidade Nova de Lisboa (ITQB NOVA), Av. da República, 2780-157 Oeiras, Portugal; 3 Centre for Functional Ecology (CEF), Department of Life Sciences, University of Coimbra, 3000-456 Coimbra, Portugal; 4 Chemical Process Engineering and Forest Products Research Centre (CIEPQPF), Department of Chemical Engineering, University of Coimbra, 3030-790 Coimbra, Portugal

**Keywords:** environmental and human health safety, essential oil, *Meloidogyne graminicola*, nematicide, rice, sustainable farming, tomato, volatile plant metabolites

## Abstract

The rice root-knot nematode (RRKN), *Meloidogyne graminicola* Golden and Birchfield 1965, is a dangerous crop pest that affects rice production on a global scale. The largest rice-producing countries struggle with the impacts of RRKN infestation, namely, underdeveloped plants and a reduction in rice grain that can reach up to 70% of crop yield. In addition, the shift to strategies of sustainable pest management is leading to a withdrawal of some of the most effective pesticides, given the dangers they pose to the environment and human health. Volatile metabolites produced by plants can offer safer alternatives. The present study characterized the nematicidal activity of volatile phytochemicals against the RRKN and compared the most active with commercial nematicides concerning their safety to the environment and human health. Rice plants were used to grow large numbers of RRKNs for direct-contact bioassays. Mortality induced by the volatiles was followed for four days on RRKN second-stage juveniles. Of the 18 volatiles tested, carvacrol, eugenol, geraniol, and methyl salicylate showed the highest mortalities (100%) and were compared to traditional nematicides using (eco)toxicological parameters reported on freely available databases. While methyl salicylate had a faster activity, carvacrol had more lasting effects. When compared to synthetic nematicides, these volatile phytochemicals were reported to have higher thresholds of toxicity and beneficial ecotoxicological parameters. Ultimately, finding safer alternatives to traditional pesticides can lower the use of damaging chemicals in farming and leverage the transition to agricultural practices with a lower impact on biodiversity.

## 1. Introduction

Plant pests are one of the most resource-draining factors in crop production, and their management is strongly reliant on pesticide use, imposing a heavy economic strain on farmers. The misuse of synthetic or hemisynthetic pesticides endangers surrounding ecosystems, plant and animal wildlife, and, more directly, soil microbial diversity [1]. Under these conditions, plant pests more easily reach extreme population numbers, are more prone to develop resistance, and rapidly spread to new locations. In the production of rice, a global staple crop, soil quality and health are becoming increasingly important due to growing concerns over food safety and climate change [2]. In the economy of Asian countries, rice production takes on a pivotal role. China and India alone account for half of global production [3]. Rice paddy fields occupy large areas and are affected by several plant pests, mainly insects and nematodes. Plant parasitic nematodes can lead to >90% yield loss in rice, particularly *Ditylenchus angustus* (Buther, 1913) Filipjev, 1936; *Aphelenchoides besseyi* Christie, 1942; *Hirschmanniella* spp. Luc and Goodey, 1964; *Heterodera oryzicola* Rao and Jayaprakash, 1978; and *Meloidogyne graminicola* Golden and Birchfield, 1965; with the latter being considered the most prevalent pest in major rice-producing countries [4]. 

The rice root-knot nematode (RRKN) *M. graminicola* was first described by Golden and Birchfield (1965), after its isolation from the roots of jungle rice (*Echinochloa colonum* L.). Since then, its presence has been reported on rice fields in almost every continent [5,6,7]. In Asia, RRKN infestation can result in yield losses of up to 70%. The rate of infection, development, and reproduction of RRKNs rises with higher temperatures and increased moisture or rainfall. This leads to changes in its abundance and geographic distribution [8]. The RRKN is considered a facultative meiotic parthenogenetic species, which means that amphimixis can occur at a low frequency. The infective second-stage juveniles (J2) enter roots near the tip, and once established become sedentary and flask-shaped, by undergoing three molts to become third (J3) and fourth (J4) juvenile stages, and finally the adult stage. The RRKN differs from other *Meloidogyne* species in that, typically, females lay eggs within the host tissue as an adaptation to the flooded conditions of rice fields. Characteristic symptoms of RRKN infestation include yellowing, stunting, and hook-like galls on the roots of rice plants [9,10]. The species *M. graminicola* is known to have a wide range of hosts besides rice, which includes cereals, grasses, weeds, and some horticultural crops [11,12].

In rice, pest management is mainly performed through cultural strategies, by crop rotation with non-hosts, e.g., cowpea, sesame, or sunflower, or maintaining flooded conditions throughout the growth cycle, to reduce new RRKN infections. However, rice production is still very dependent on pesticide use, and in Asia, non-fumigant nematicides, e.g., carbofuran or oxamyl, and fumigants, e.g., 1,3-dichloropropene, are liberally used for RRKN control [13]. In the European Union, over 30% of the analyzed rice samples contain residues of pesticides, and, in almost 7%, pesticide concentrations were found above the maximum residue level allowed [14]. The EU expects to reduce the use of pesticides in its territory by 50% in the coming years, and many of the most dangerous chemicals, e.g., carbofuran, oxamyl, and 1,3-dichloropropene, have already been banned and/or substituted by less environmentally damaging alternatives [15]. However, concerns over their efficacy have been rising among farmers. Consequently, in the past few years, a strong pressure for the development of safer biopesticides has prompted research to screen active plant extracts for nematicidal activity, from which promising results have been obtained for essential oils (EOs) [16,17]. EOs are complex mixtures of volatile compounds, mainly composed of varying amounts of mono-, sesqui-, and/or diterpenes and phenylpropanoids, and in some cases high amounts of compounds from other chemical classes [18]. These compounds can have different degrees of biological activity against plant parasitic nematodes and can often display synergistic and/or antagonistic interactions, and thus contribute positively and/or negatively, respectively, to the nematicidal activity of EOs [19]. The chemical profiling of EOs has become an easy and affordable technique. Hence, research on EOs for the development of biopesticides often resorts to screening the main EO compounds to uncover the origin of nematicidal activity. These active components can often be used as precursors of biopesticides with optimized activity. Biopesticides are considered pesticides derived from natural materials, for example, metabolites, extracts, or parts of plants or microorganisms. Biochemical biopesticides are naturally occurring compounds able to control pests through non-toxic mechanisms. Many EOs and single EO compounds are now approved active substances of biopesticides [20]. However, care must be taken, since EO composition can easily vary depending on the genetic makeup and environmental conditions of the plant used, and non-approved compounds can then have a strong presence [18]. Also, while a single EO compound can show high activity, the complex mixture of volatiles that compose an EO can interact synergistically or antagonistically, influencing each component’s activity [21,22].

Against RKNs, EOs rich in the monoterpenes carvacrol, citronellal, *p*-cymene, geraniol, linalool, or α-pinene and the phenylpropanoids anethole or eugenol have shown high activity. The main mechanisms of action associated with these compounds are disruption of the nervous system (for α-pinene, linalool, and anethole), alteration of cellular processes (for geraniol, eugenol, and anethole), and disruption of plasma membrane permeability (for carvacrol, citronellal, and eugenol) in J2 and adult females [23].

The present work is aimed at screening for the nematicidal activity of volatile compounds commonly found in EOs against the RRKN. The most active volatiles were compared to commercial nematicides on their reported toxicity thresholds for model organisms and predicted environmental fate [24]. This approach contributes to the development of sustainable alternatives to the synthetic nematicides used in modern farming.

## 2. Materials and Methods

### 2.1. Chemicals

The compounds tested were pure commercial standards of EO components and were acquired from Sigma-Aldrich (St. Louis, MO, USA), with the exception of geranyl acetone, which was acquired from TCI (Tokyo, Japan) (Table 1). The commercially available nematicide oxamyl (AFROMYL, Epagro, Alverca do Ribatejo, Portugal) was also tested. For the direct-activity bioassays, HPLC-grade methanol, acquired from Fisher Chemicals (Newington, NH, USA), was used to prepare stock solutions of the pure compounds.

### 2.2. Rice Root-Knot Nematode Growth

*Meloidogyne graminicola* was kindly provided by the European Union Reference Laboratory (EURL) for plant parasitic nematodes, in the form of root galls. It is regularly maintained in rice, *Oryza sativa* L. var. Ariete, the most commonly grown rice variety in Portugal [26], as a reference isolate at the Plant Nematology Lab of the National Institute for Agrarian and Veterinary Research (INIAV, I.P.), in Oeiras, Portugal. Seeds were germinated at 26 ± 1 °C on hydrated filter paper and then transplanted into dark plastic pots (0.5 L) filled with a sterilized 2:1 mixture of sand and biological substrate (SIRO, Dublin, Ireland). A wet environment was maintained by daily watering, and fertilizer was applied once a week (0.5 g/L; Green House Feeding (Hybrids); NPK: 15:7:22). After ca. 7 days, each host seedling was inoculated with 200 RRKNs J2 and maintained in a growth chamber at 26 ± 1 °C. After ca. 45 days, host roots already displayed fully developed root galls with visible egg masses. The plants were harvested, and the root systems washed [27]. The eggs were isolated from the roots by mixing with a 0.52% (*v*/*v*) NaOCl solution [28] and were hatched in moist chambers at 25 ± 1 °C. The RRKN J2s were counted by sampling five aliquots of 100 µL from the 5 mL solution in which the eggs were hatched. The quantification of nematodes and/or assessment of survival rates was performed with an Olympus SZX12 (Tokyo, Japan) stereomicroscope.

### 2.3. Nematicidal Activity

To screen for nematicidal activity against the RRKN, stock solutions of the chemical standards were prepared with methanol at 20 mg/mL and maintained at −20 °C. Aqueous suspensions of RRKNs were prepared containing ca. 600 ± 60 J2/mL, and aliquots of 95 µL were pipetted per well on 96-well microtiter plates (Carl Roth GmbH + Co. KG, Karlsruhe, Germany). Next, 5 µL of compound stock solution was added to the J2 suspension to obtain a final concentration of 1 mg/mL in the wells [29]. Two separate bioassays were performed, each with at least 6 replicates per compound. To assess natural mortality and the mortality induced by methanol, blank and control wells were also prepared by adding 5 µL of ultrapure water or methanol, respectively. Finally, the suspensions were mixed by setting the microtiter plate in an orbital shaker (IKA labortechnik, Staufen, Germany) at 800 cycles/min for 1 min. The plates were then covered and maintained in darkness at 25 ± 1 °C in an orbital shaker at 50 r.p.m. for 96 h. The live and dead nematodes were counted at 1, 12, 24, 48, 72, and 96 h, as described above. For immobile J2s, mortality was ascertained by mechanically probing for movement.

### 2.4. Environmental Safety and Toxicity to Mammals

Environmental safety and toxicity to mammals were estimated for the compounds with the highest nematicidal activity by determining their likely environmental fate and the ecotoxicological and toxicological parameters reported by recognized databases [24,30]. A predictive equilibrium criterion model [31] was used to compare the estimated environmental fate of carvacrol, eugenol, geraniol, and methyl salicylate with that of the synthetic nematicides oxamyl (a carbamate of systemic activity) and methyl isothiocyanate (as the active compound of the fumigant nematicides metam sodium and dazomet). The Level I Mackay fugacity model beta version 4.31 (Trent University, Peterborough, ON, Canada) [32] was employed to determine percentages of predicted environmental distribution (PED) in the air, water, soil, and sediment environmental compartments. The Level I model was set to assume the introduction of an illustrative 100,000 kg into a closed system under steady-state and equilibrium conditions at 25 °C. The experimental data required on the chemical properties of each compound, namely, molecular mass (g/mol), melting point (°C), vapor pressure (Pa), solubility in water (mg/L), air–water partition coefficient/Henry’s law constant (Pa·m^3^/mol), *n*-octanol–water partition coefficient (log Kow), and soil organic carbon–water partition coefficient (Koc), were obtained from the publicly available PubChem online database [33] and PPDB: the Pesticide Properties Database [34] (Supplementary Table S1).

The toxicological and ecotoxicological experimental data on the nematicidal compounds and the synthetic nematicides oxamyl and methyl isothiocyanate were accessed on PubChem [33], PPDB: the Pesticide Properties Database [34], and ECHA, the European Chemicals Agency [35]. The predicted toxicological and ecotoxicological parameters were obtained with the Toxicity Estimation Software Tool (TEST) version 5.1.2. and EPI (Estimation Programs Interface) Suite software version 4.11. developed by the U.S. Environmental Protection Agency [36,37].

### 2.5. Data Treatment and Statistical Analysis

The numbers of live and dead nematodes were converted to mortality percentages through Formula (1),

mortality% = (no. of dead nematodes/total no. of nematodes) × 100,(1)

For each compound, the Schneider–Orelli Formula (2) was used to obtain corrected mortality percentages by subtracting the mortality in the control wells [21]:

corrected mortality% = [(mortality% in treatment − mortality% in control)/(100 − mortality% in control)] × 100,(2)

At each time point, the compound’s nematicidal strength was classified as either complete (100%), strong (80–99%), moderate (61–79%), weak (40–60%), or low/inactive (<40%) [38]. The statistical analysis was performed with Version 2019 of Origin Graphing and Analysis software version 10.15 (OriginLab, Northampton, MA, USA) [39]. Statistical significance was determined with one-way ANOVA, and individual means were compared using Tukey’s post hoc test with *p* < 0.05, the Shapiro–Wilk test ensured data normality, and the Browns–Forsythe test was used for homoscedasticity. The half-maximal effective concentration (EC_50_) values were determined through a nonlinear regression analysis by fitting a log-logistic equation [21] to the corrected mortality values (y) plotted against the compound concentration values (x), i.e., fitting the dose–response Equation (3),

y = C + (D − C)/1 + exp {b [log (x) − log (EC_50_)]}, (3)

where C is the lower and D is the upper limit of the sigmoidal curve, b is the slope, and EC_50_ is the EO concentration that induces a response halfway between the lower and upper limits. The upper (D) and lower (C) limits were set to 0 and 100%, respectively. The determination of the lowest maximal effective concentration (EC_100_) was performed by solving the curve equation to the first fitted y value of 100% mortality. The results are presented as the average and standard error of 10 replicates.

## 3. Results

### 3.1. Nematicidal Activity of the Volatiles 

Rice plants produced large numbers of RRKNs to be used in the screening of the nematicidal activity of the volatile phytochemicals in comparison to the pesticide oxamyl. Most of the compounds tested against the RRKN J2s revealed low activity (<40%). The monoterpene hydrocarbons were inactive, while for the oxygen-containing monoterpenes, the results varied. Of these, the most active were the phenol carvacrol and the alcohol geraniol, inducing complete mortality at all tested points of the time course study (Table 2). 

The aldehyde citronellal and the alcohol menthol showed strong activity in the beginning (95%, after 1 h, for citronellal, and 87–90%, between 1 and 24 h, for menthol) but lost their activity by the end of the time course study. The alcohol α-terpineol showed a peak of activity at 24 h but then decreased until the end of the time course study. The remaining oxygen-containing monoterpenes and the sesquiterpene hydrocarbon *trans*-β-caryophyllene were inactive (<40%). For the phenylpropanoids, only eugenol showed strong activity at 24 h and onwards; however, at 1 and 12 h, mortality was 30 and 65%, respectively. Methyl salicylate showed a peak of activity at 24 h and decreased from then onwards but maintained a strong activity throughout (>80%). The ketone 2-undecanone was inactive against the RRKN (Supplementary Table S2). The activity of oxamyl (the active compound of a commercial nematicide) was very high but not complete at the highest concentration tested. However, its mortality was seen to increase with time of exposure (73.9 ± 1.3, in the first hour, to 95.7 ± 1.1, after 96 h) suggesting a long-term influence on the RRKN.

The most active volatiles, i.e., carvacrol, eugenol, geraniol, and methyl salicylate, were tested at lower concentrations to determine their toxicological parameters (Table 3). Methyl salicylate showed the lowest values for EC_50_ and EC_100_ at 12 and 24 h, suggesting stronger activity immediately after application. The activity of carvacrol was highest after 48, 72, or 96 h; at these points in the time course study, the lowest EC_50_ and EC_100_ values were obtained, indicating that, unlike methyl salicylate, carvacrol had a higher effect the longer the contact with the RRKN. The phenylpropanoid eugenol and the monoterpene geraniol showed intermediate activities, reaching their lowest EC_50_ and EC_100_ values at 24 h of direct contact with the RRKN. 

### 3.2. Toxicity to Mammals

The potential for toxicity to mammals was assessed for the nematicidal volatiles in comparison to the synthetic nematicides oxamyl, a carbamate of systemic activity, and methyl isothiocyanate, the volatile active compound released after the environmental degradation of the fumigant nematicides metam sodium or dazomet. The experimental LD_50_ values reported on freely available online databases for oral toxicity tested on rats were higher for carvacrol, eugenol, geraniol, and methyl salicylate (810 to >4000 mg/kg) than for oxamyl or methyl isothiocyanate (3 and 147 mg/kg, respectively) (Table 4). For dermal toxicity, the lowest values were reported for methyl salicylate and methyl isothiocyanate (700 and 1290 mg/kg, respectively); the highest was reported for oxamyl (5000 mg/kg), and carvacrol, eugenol, and geraniol were reported to have LD_50_ values between 2000 and 2700 mg/kg. Using specialized software, the volatiles carvacrol and eugenol were predicted to exert developmental toxicity, along with the synthetic nematicides oxamyl and methyl isothiocyanate, but geraniol and methyl salicylate were predicted not to have this effect (Table 4). Concerning their potential for mutagenicity, only the synthetic nematicides were predicted to possess a high likelihood. 

### 3.3. Environmental Safety

To evaluate the potential for environmental damage, the reported chemical properties of the most successful volatiles and the commercial nematicides oxamyl and methyl isothiocyanate were used to predict their potential environmental distribution, their persistence in water compartments, and removal through wastewater treatment. Carvacrol was predicted to remain mainly in the soil environmental compartment (73%), followed by eugenol (41%), methyl salicylate (21%), and geraniol (16%) (Table 5). Inversely, geraniol showed the highest affinity to the water compartment (83%), followed by methyl salicylate (77%), eugenol (56%), and, lastly, carvacrol (23%). Due to its high solubility in water, oxamyl was predicted to remain mostly in the water environmental compartment, while methyl isothiocyanate had a high affinity with the air compartment (due to its low molecular weight and high volatility) as well as the water environmental compartment. Persistence was predicted to be higher for oxamyl and lower for geraniol and methyl salicylate. The volatilization from water, predicted as the half-life of the compound in water, was lower for geraniol and methyl salicylate, for both the river and lake models, followed by methyl isothiocyanate, eugenol, and carvacrol, but was highest for oxamyl (over 8000-fold) (Table 5). The expected removal from wastewater treatment was almost complete for geraniol and methyl salicylate, mainly through volatilization into the air, but lower for the remaining compounds (2 to 6%, mainly through sludge adsorption and release into the air, as was the case for methyl isothyocianate). 

Online databases report experimental ecotoxicological data previously obtained by legitimate sources. By comparing the half-maximal effective doses (EC_50_) reported for the most active volatiles with those reported for the synthetic nematicides, methyl isothiocyanate stood out for its extreme toxicity to invertebrates, fish, and algae model organisms (Table 6). Despite its volatility, this nematicide appears to be highly damaging to ecosystem biodiversity. Oxamyl also showed lower EC_50_ values than the tested phytochemical volatiles, with the exception of the activity reported for eugenol on fish model species (1 mg/L for the rainbow trout *Oncorhynchus mykiss* (Walbaum, 1792)). Overall, the volatiles appear to have a lower impact on non-target organisms than the commonly used nematicides.

## 4. Discussion

In the EU, the Green Deal initiative set out activities to reduce the risks and the impact of pesticide use on human health and the environment, with the aim of reducing the use of chemical pesticides by 50% [40]. Tapping into the phytochemical arsenal of plants has already yielded strong nematicidal products, e.g., with neem or garlic extracts or their main compounds [41,42]. However, sustainable alternatives are still underused.

From the direct contact bioassays performed in the present study, four volatiles, commonly found in plant EOs, induced strong nematicidal activities against the RRKN. The monoterpenes carvacrol (a phenol) and geraniol (an alcohol), and the phenylpropanoids eugenol and methyl salicylate were able to interfere with J2 motility at 1 mg/mL, while for methyl salicylate, this activity was transient; after 24 h, the RRKNs showed movement again, whereas, with the other phytochemicals, the nematodes were immobilized throughout. When tested at lower concentrations, eugenol and geraniol began to lose full activity (complete mortality, i.e., LC_100_) before carvacrol and methyl salicylate. Eugenol and geraniol showed their best results at 24 h of direct contact. However, carvacrol showed the lowest EC_50_ values at the end of the time course study, while methyl salicylate at the beginning, suggesting that carvacrol can be a long-duration contact nematicide, while methyl salicylate acted more immediately. 

There is still not much in the literature dealing with the activity of phytochemicals against RRKNs in comparison with other *Meloidogyne* species. In a study that screened the EOs from *Syzygium aromaticum*, *Cymbopogon flexuosus,* and *C. martinii* and their respective main volatiles, namely, eugenol, citral, and geraniol, against the RRKN, the activities observed at concentrations that varied from 0.5 to 0.03 mg/mL were not substantially different between the EOs and their main compound, indicating that these were responsible for the nematicidal activity [43]. In this study, the activity of citral was comparable to that of eugenol and geraniol, unlike what was observed in the present study. This may be due to different ratios of the geometric isomers that constitute it, geranial (*trans*-citral) and neral (*cis*-citral), which can change in different environmental conditions (high temperature, light, and oxygen), especially since geranial is chemically more unstable than neral [44]. These isomers may even have different activities towards the RRKN, as was previously observed for the pinewood nematode, *Bursaphelenchus xylophilus* (Steiner and Buhrer, 1934) Nickle, 1981, where geranial showed a five-fold higher nematicidal activity than neral [16,45]. Against *M. javanica* (Treub, 1885) Chitwood, 1949, citral also showed very low activity (35% of mortality) at a four-fold higher concentration than the highest used in the present study, while for *M. ethiopica* Whitehead, 1968, it induced complete mortality when bioassayed at 1 mg/mL for 24, 48, 72, and 96 h of direct contact [46]. The compound(s) used to solubilize volatiles in the aqueous suspensions of nematodes can also contribute to differences in activity as previously reported [47]. For eugenol, reported EC_50_ values varied between 0.3 and 0.4 mg/mL at 24 h direct contact, 0.2 and 1.2 mg/mL at 48 h, and 0.1 mg/mL at 72 h and 96 h [48,49,50,51], for *M. incognita* (Kofoid and White, 1919) Chitwood, 1949 and for *M. javanica*. For geraniol, reported EC_50_ values were 0.2 mg/mL at 24 h, 0.2 and 1.2 mg/mL at 48 h, and 0.1 mg/mL at 96 h [48,50] for *M. incognita* and *M. javanica*. Carvacrol has been previously tested against *M. incognita* and *M. javanica,* yielding EC_50_ values that varied between 0.01 and 0.3 mg/mL, at 24 h direct contact, 0.2 mg/mL at 48 h, and 0.1 mg/mL at 96 h [48,52,53]. Methyl salicylate has been less studied in direct contact bioassays. Using the methyl salicylate-rich EO of *Filipendula ulmaria* (L.) Maxim. (85%), very strong activity was reported against the hatching of eggs of *M. chitwoodi* Golden, O’Bannon, Santo, and Finley, 1980 (EC_50_ of 0.032 µL/mL). Methyl salicylate appears to be involved in RKN chemotaxis. In a study analyzing the host location of *Capsicum annum* L. cultivars by *M. incognita* J2, methyl salicylate was seen to exert the highest positive chemotaxis, and thymol, an isomer of carvacrol, the highest negative chemotaxis [54]. In tomato, *S. lycopersicum*, methyl salicylate and methyl dihydrojasmonate appear to influence the attraction of *M. javanica* J2 to susceptible roots, despite the root emission of attraction monoterpenes [55]. Their influence on RKN motility and infection appears to be dependent on the presence of other volatiles or on the type of host plant [54,55]. Methyl salicylate can be easily hydrolyzed under biological conditions to salicylic acid [56], a product that has also been seen to modulate the infection of hosts by the RKN [57,58]. The potential dual activity of methyl salicylate, as attractant and nematicide, can contribute to the development of a more effective bionematicide by being used as an adjuvant, or even exploring its synergistic interaction with the other volatile nematicides identified, carvacrol, geraniol, or eugenol, since volatile phytochemicals are known to often display these interactions [19,22]. 

The use of these phytochemical volatiles could provide greater safety to human health since their reported oral toxicity threshold values (LD_50_) are much higher than those reported for the synthetic nematicides. Also, they are predicted to have no mutagenic activity, and, for toxicity to development, only carvacrol and eugenol were predicted toxicants. In contrast, the physicochemical characteristics of the synthetic nematicides led to their prediction as mutagenic and developmental toxicants. In effect, carvacrol, eugenol, and methyl salicylate are classified under the GHS Hazard Classification as irritants, and geraniol as an irritant and corrosive, while the synthetic nematicides are classified as irritant, corrosive, acutely toxic, flammable and/or environmental hazards [33,59]. These phytochemical volatiles are currently mostly used in the food industry as additives or flavoring agents, or in cosmetics as perfumes, thus their major hazards are skin irritation, allergic reactions, or eye irritation at very high amounts, also being harmful if swallowed. 

Their selection for the development of bionematicides can be further supported by their environmental safety. While oxamyl was predicted to have a higher affinity to the water compartment, with higher persistence and lower volatilization or low removal, the volatile phytochemicals were predicted to be less persistent and have less affinity to water bodies, being easily volatilized, in the case of geraniol and methyl salicylate. Given its chemical composition, methyl isothiocyanate was predicted to have the highest affinity to the air compartment, resulting in a higher predicted volatilization and lower persistence; however, the reported thresholds for toxicity to aquatic organisms are very low, indicating a strong ecotoxicity. An accidental release of 72,000 L of the pesticide Metam, whose active ingredient is methyl isothiocyanate, into the Upper Sacramento River, California, USA, resulted in changes in the riverbed’s microbial community that persisted a full year after the spill, indicating a physiologic accommodation or selection for resistance at the population, species, and/or community level [60]. For the volatile phytochemicals tested, reported toxicity threshold values were higher, suggesting lower toxicity. For geraniol and methyl salicylate, these lower toxicity values for non-target organisms, combined with high volatility, suggest their potential valuable contribution to the development of a sustainable bionematicide. In fact, geraniol and eugenol are now used as agrochemical pesticides in integrated management, in minimum-risk pesticides for combating insect pests, alongside highly bioactive EOs (e.g., mint, cinnamon, and lemongrass), which can make their acceptance easier for end-users and also facilitate the legal approval of any bionematicide created. 

## 5. Conclusions

Modern farming must shift to lower environmental impact if it expects to reach sustainability. Development of new biopesticides with directed activity relies on active research of novel active components. The volatile phytochemicals carvacrol, eugenol, geraniol, and methyl salicylate were shown to have high nematicidal properties against the RRKN, providing good candidates for the development of bionematicides to be used in rice production. Their chemical characteristics grant them lower ecotoxicological properties and less harmful environmental impacts than the synthetic nematicides used nowadays. Their integration into modern pest management strategies can lead to a reduction in the use of harmful pesticides and a lower influence on biodiversity. Volatiles can be the base for safer biopesticides to replace traditional nematicides in farming and leverage the transition to agricultural practices with a lower impact on biodiversity.

## Figures and Tables

**Table 1:** Commercial standards used for direct-contact bioassays, their purity (%), chemical functional group, and an example of an essential oil (EO) with reported high amounts in its composition (%).

Compound	Functional Group	Purity (%)	Plant EO with High Amounts (%) ^1^
**Monoterpene hydrocarbons**			
*p*-Cymene	-	99	*Origanum saccatum* (74) [25]
α-Pinene	-	98	*Eucalyptus pauciflora* (82) [21]
*γ*-Terpinene	-	97	*Crithmum maritimum* (68) [25]
**Oxygen-containing monoterpenes**			
Carvacrol	phenol	≥98	*Thymbra capitata* (69) [21]
Citronellal	aldehyde	≥95	*Corymbia citriodora* (35) [21]
Citral ^2^	aldehyde	≥96	*Cymbopogon citratus* (71) [21]
Geraniol	alcohol	≥97	*Cymbopogon martinii* (93) [25]
Geranyl acetone	ketone	≥96	*Ziziphus spina-christi* (14) [25]
Linalool	alcohol	≥97	*Coriandrum sativum* (92) [25]
Menthol	alcohol	99	*Mentha arvensis* (82) [25]
Pulegone	ketone	98	*Mentha cervina* (86) [21]
α-Terpineol	alcohol	≥96	*Thymus zygis* (60) [21]
Terpinen-4-ol	alcohol	≥95	*Cryptomeria japonica* (24) [21]
**Sesquiterpene hydrocarbon**			
*trans*-β-Caryophyllene	-	≥80	*Bidens gardneri* (77) [25]
**Phenylpropanoids**			
*trans*-Anethole	phenol	99	*Foeniculum vulgare* (73) [21]
Eugenol	phenol	98	*Syzygium aromaticum* (92) [21]
**Salicylate**			
Methyl salicylate	ester	≥98	*Polygala cyparissias* (97) [25]
**Methyl ketone**			
2-Undecanone	ketone	99	*Ruta graveolens* (94) [21]

^1^ Plant species reported to contain high amounts of the compound, as potential sources of EOs for the formulation of biopesticides. ^2^ Citral is a mixture of the two geometric stereoisomers, geranial (*trans*-citral) and neral (*cis*-citral).

**Table 2:** Volatile phytochemicals with activity (≥40% mortality) tested at 1 mg/mL on second-stage juveniles of the rice root-knot nematode, after 1, 12, 24, 48, 72, or 96 h of direct contact. Values are average ± standard error of 10 repetitions. Different letters indicate statistically significant differences (*p* < 0.05) based on Tukey’s test, between time points for each compound.

RKN Mortality (%) ^1^	Bioassay Time (h)					
	1	12	24	48	72	96
**Oxygen-containing monoterpenes**						
Carvacrol	100.0 ± 0.0 a	100.0 ± 0.0 a	100.0 ± 0.0 a	100.0 ± 0.0 a	100.0 ± 0.0 a	100.0 ± 0.0 a
Citronellal	94.9 ± 0.2 a	47.5 ± 0.1 b	0.0 ± 0.0 d	1.1 ± 0.3 cd	2.1 ± 0.4 c	2.0 ± 0.5 c
Geraniol	100.0 ± 0.0 a	100.0 ± 0.0 a	100.0 ± 0.0 a	100.0 ± 0.0 a	100.0 ± 0.0 a	100.0 ± 0.0 a
Menthol	87.0 ± 0.8 a	88.7 ± 0.8 a	90.3 ± 0.8 a	57.7 ± 1.4 b	39.1 ± 1.0 c	6.9 ± 0.4 d
α-Terpineol	48.9 ± 0.9 c	62.3 ± 0.6 b	75.8 ± 1.1 a	31.3 ± 0.6 d	2.1 ± 0.4 e	1.8 ± 0.5 e
**Phenylpropanoid**						
Eugenol	29.9 ± 0.9 c	65.0 ± 0.5 b	100.0 ± 0.0 a	100.0 ± 0.0 a	100.0 ± 0.0 a	100.0 ± 0.0 a
**Salicylate**						
Methyl salicylate	92.2 ± 0.3 c	96.1 ± 0.2 b	100.0 ± 0.0 a	99.3 ± 0.2 a	97.0 ± 0.5 b	81.5 ± 0.7 d
**Pesticide**						
Oxamyl ^2^	73.9 ± 1.3 c	81.2 ± 1.2 b	83.3 ± 1.4 b	92.7 ± 1.8 a	95.3 ± 1.2 a	95.7 ± 1.1 a

^1^ For activity of compounds with corrected mortality < 40%, see Supplementary Table S2. ^2^ The active compound of the pesticide Afromyl.

**Table 3:** Half-maximal effective concentration (EC_50_, in mg/mL) and lowest maximal effective concentration (EC_100_, in mg/mL) of carvacrol, eugenol, geraniol, and methyl salicylate on *M. graminicola*, obtained by fitting a dose–response sigmoidal curve. EC_50_ values are presented as average ± standard error and EC_100_ as average with upper and lower 95% confidence intervals. The goodness-of-fit for the sigmoidal curve was evaluated through adjusted R^2^.

	Carvacrol	Eugenol	Geraniol	Methyl Salicylate
**12 h**				
EC_50_ (mg/mL)	0.152 ± 0.001	0.416 ± 0.013	0.655 ± 0.012	0.103 ± 0.003
EC_100_ (mg/mL)	0.56 (0.52–0.58)	>1	0.93 (0.85–0.98)	0.55 (0.25–0.60)
*p* ^1^	14.57 ± 0.41	2.09 ± 0.121	5.05 ± 0.38	13.42 ± 1.61
Goodness-of-fit (R^2^)	0.998	0.932	0.992	0.957
**24 h**				
EC_50_ (mg/mL)	0.161 ± 0.001	0.182 ± 0.001	0.386 ± 0.005	0.115 ± 0.001
EC_100_ (mg/mL)	0.50 (0.48–0.51)	0.76 (0.74–0.78)	0.78 (0.72–0.81)	0.40 (0.18–0.44)
*p*	17.82 ± 0.38	10.30 ± 0.16	15.03 ± 0.67	20.93 ± 3.99
Goodness-of-fit (R^2^)	0.999	0.997	0.999	0.998
**48 h**				
EC_50_ (mg/mL)	0.150 ± 0.001	0.230 ± 0.002	0.398 ± 0.003	0.189 ± 0.005
EC_100_ (mg/mL)	0.22 (0.09–0.29)	0.83 (0.78–0.87)	0.83 (0.80–0.84)	0.49 (0.33–0.52)
*p*	80.75 ± 2.40	6.63 ± 0.22	13.99 ± 0.43	19.65 ± 1.60
Goodness-of-fit (R^2^)	0.999	0.992	0.999	0.978
**72 h**				
EC_50_ (mg/mL)	0.147 ± 0.005	0.277 ± 0.001	0.462 ± 0.062	0.229 ± 0.044
EC_100_ (mg/mL)	0.23 (0.09–0.30)	0.80 (0.77–0.82)	0.78 (0.40–0.87)	0.39 (0.16–0.47)
*p*	69.86 ± 9.17	7.65 ± 0.16	18.89 ± 0.62	36.57 ± 0.05
Goodness-of-fit (R^2^)	0.999	0.998	0.999	0.948
**96 h**				
EC_50_ (mg/mL)	0.155 ± 0.002	0.495 ± 0.001	0.514 ± 0.060	0.246 ± 0.06
EC_100_ (mg/mL)	0.21 (0.11–0.26)	0.91 (0.90–0.93)	0.78 (0.43–0.92)	0.38 (0.17–0.47)
*p*	114.53 ± 4.15	4.75 ± 0.08	15.15 ± 0.22	30.19 ± 0.06
Goodness-of-fit (R^2^)	0.999	0.999	0.998	0.929

^1^ Values for the slope (*p*) are shown for comparison purposes.

**Table 4:** Experimental acute toxicity (oral and dermal) thresholds for mammals (median lethal dose, LD_50_, mg/kg) reported in the freely available PubChem online database [33] and PPDB: the Pesticide Properties Database [34]. Predicted developmental toxicity and mutagenicity levels obtained with the Toxicity Estimation Software Tool (TEST) [36].

Compounds	Oral Toxicity LD_50_ (mg/kg)	Dermal Toxicity LD_50_ (mg/kg)	Predicted ^1^ Developmental Toxicity	Predicted ^1^ Mutagenicity
Carvacrol	810	2700	Toxicant	Negative
Eugenol	>1930	2000	Toxicant	Negative
Geraniol	>4000	2000	Non-toxicant	Negative
Methyl Salicylate	887	700	Non-toxicant	Negative
Oxamyl	3	5000	Toxicant	Positive
Methyl Isothiocyanate	147	1290 ^2^	Toxicant	Positive

^1^ Parameters were estimated using the nearest neighbor method, where prediction is based on the values of three of the most similar chemicals. ^2^ Value reported for rabbits.

**Table 5:** Predicted environmental distribution (PED, %) in the environmental compartments of air, water, soil, and sediments, using the Mackay fugacity model [31,37], predicted volatilization from water (using river and lake models) [37] and predicted removal in wastewater treatment, obtained by the EPI Suite Estimation Program Interface [37] and based on the experimental chemical properties reported in online databases [33,34] for the volatiles compared to oxamyl and methyl isothiocyanate.

Environmental Fate	Carvacrol	Eugenol	Geraniol	Methyl Salicylate	Oxamyl	Methyl Isothiocyanate
Air (%)	2	2	0	1	0	74
Sediments (%)	2	1	0	0	0	0
Soil (%)	73	41	16	21	3	0
Water (%)	23	56	83	77	97	26
Persistence (h)	429	461	63	83	1150	305
**Volatilization from water ^1^**						
Model river (half-life in h)	444	378	1	1	4 × 10^6^	9
Model lake (half-life in h)	4949	4235	118	117	4 × 10^7^	172
**Removal in wastewater treatment ^2^**						
Total removal (%)	6	2	100	100	2	5
Biodegradation (%)	0	0	0	0	0	0
Sludge adsorption (%)	6	2	2	0	2	2
Release into the air (%)	0	0	97	99	0	3

^1^ obtained using WVOLWIN module for the estimate of the rate of volatilization of a chemical from rivers and lakes [37]; ^2^ obtained using STPWIN module to predict the removal of a chemical in a typical activated sludge-based sewage treatment plant [37].

**Table 6:** Reported half-maximal effective dose (EC_50_, in mg/L) values for invertebrates, fish, and algae of the volatiles compared to oxamyl and methyl isothiocyanate. Values were retrieved from online databases [33,34,35].

Reported EC_50_ (mg/L)	Carvacrol	Eugenol	Geraniol	Methyl Salicylate	Oxamyl	Methyl Isothiocyanate
Invertebrates (48 h) ^1^	6	>10	12	1501	0.3	0.1
Fish (96 h) ^2^	6	1	16	28	3	0.1
Algae (96 h) ^3^	4	15	48	2	1	0.6

^1^ The values presented were reported for the invertebrate model *Daphnia magna* (Straus, 1820); ^2^ The values presented were reported for methyl salicylate activity on *Pimephales promelas* (Rafinesque, 1820), for carvacrol activity on *Brachydanio rerio* (F. Hamilton, 1822), and for geraniol, eugenol, or oxamyl activity on *Oncorhynchus mykiss*. ^3^ The values presented were reported for the model algae species *Pseudokirchneriella subcapitata* (Korshikov) hindák.

## Data Availability

The raw data supporting the findings of this study are available from the corresponding author (Jorge M. S. Faria) upon reasonable request.

## Supplementary Materials

The following supporting information can be downloaded at: https://www.mdpi.com/article/10.3390/plants13152046/s1, Table S1. Physical and chemical properties of the most active compounds and traditional nematicides required to perform the Level I Mackay fugacity model [molecular mass (g/mol), melting point (°C), vapor pressure (Pa), solubility in water (mg/L), air–water partition coefficient or Henry’s Law constant (Pa·m^3^/mol), *n*-octanol–water partition coefficient (log Kow), and soil organic carbon–water partition coefficient (Koc). Table S2. Nematicidal activity of volatile phytochemicals tested at 1 mg/mL against second-stage juveniles of the rice root-knot nematode, after 1, 12, 24, 48, 72, or 96 h of direct contact. Values are average ± standard error of 10 repetitions. Different letters indicate statistically significant differences (*p* < 0.05) based on Tukey’s test, between time points for each compound. Figure S1. Half-maximal effective concentration (EC_50_, in mg/mL) (a) and lowest maximal effective concentration (EC_100_, in mg/mL) (b) of carvacrol, eugenol, geraniol, and methyl salicylate on *M. graminicola*, obtained by fitting a dose–response sigmoidal curve. Slope (*p*) values are presented for comparison purposes (c).
