# Supplementary material for: Nematicidal Activity of Volatiles against the Rice Root-Knot Nematode and Environmental Safety in Comparison to Traditional Nematicides
Source: Plants (Basel). 2024 Jul 25;13(15):2046. doi: 10.3390/plants13152046 (PMC11314546; doi:10.3390/plants13152046)
Supplement: Supplementary file 1 [file plants-13-02046-s001.zip › plants-3078424-supplementary.pdf]

Supplementary Materials

Nematicidal Activity of Volatiles against the Rice Root-Knot Nematode and Environmental Safety in Comparison to Traditional Nematicides

Jorge M. S. Faria [1,2,*], Leidy Rusinque [1,2,3,4] and Maria L. Inácio [1,2]

[1] INIAV, I.P., National Institute for Agrarian and Veterinary Research, Quinta do Marquês, 2780-159 Oeiras, Portugal; leidy.rusinque@iniav.pt (L.R.); lurdes.inacio@iniav.pt (M.L.I.)
[2] GREEN-IT Bioresources for Sustainability, Instituto de Tecnologia Química e Biológica, Universidade Nova de Lisboa (ITQB NOVA), Av. da República, 2780-157 Oeiras, Portugal
[3] Centre for Functional Ecology (CEF), Department of Life Sciences, University of Coimbra, 3000-456 Coimbra, Portugal
[4] Chemical Process Engineering and Forest Products Research Centre (CIEPQPF), Department of Chemical Engineering, University of Coimbra, 3030-790 Coimbra, Portugal
* Correspondence: fariajms@gmail.com

Supplementary Table S1. Physical and chemical properties of the most active compounds and traditional nematicides, required to perform the Level I Mackay Fugacity Model [molecular mass (g/mol), melting point (°C), vapor pressure (Pa), solubility in water (mg/L), air–water partition coefficient or Henry's Law constant (Pa.m³/mol), n-octanol/water partition coefficient (logKow) and soil organic carbon/water partition coefficient (Koc).

Chemicals	CAS Number	Molecular Mass (g/mol)	Melting Point (°C)	Vapor Pressure (Pa)	Solubility in H_2O (mg/L)	Henry's Law Constant ($Pa.m^3/mol$)	$logK_{OW}$ (Unitless)	K_{OC} (Unitless)
Carvacrol	499-75-2	150.22	1.0	3.1	1250	0.371	3.33	1467
Geraniol	106-24-1	154.25	-15.0	4.0	100	$1.533x10^{-3}$	3.56	90
Eugenol	97-53-0	164.20	-10.0	3.0	2460	0.195	2.49	340
Methyl Salicylate	119-36-8	152.15	-8.6	4.6	7400	0.095	2.55	128
Oxamyl	23135-22-0	219.26	98.5	$1.8x10^{-5}$	148100	$2.670x10^{-7}$	-0.44	15
Methyl Isothiocyanate	556-61-6	73.12	36.0	471.9	7600	14.200	0.94	9

Supplementary Table S2. Nematicidal activity of volatile phytochemicals tested at 1 mg/mL against second stage juveniles of the rice root-knot nematode, after 1, 12, 24, 48, 72 or 96 h of direct contact. Values are average±standard error of 10 repetitions. Different letters indicate statistically significant differences ($p < 0.05$) on the basis of Tukey's test, between time-points for each compound.

RKN mortality (%)	Bioassay time (h)					
	1	12	24	48	72	96
**Monoterpene hydrocarbons**						
p-Cymene	0.0±0.0b	0.0±0.0b	0.0±0.0b	1.8±0.5a	2.1±0.4a	1.8±0.5a
α-Pinene	12.4±0.7a	6.2±0.3b	0.0±0.0c	1.6±0.4c	1.5±0.3c	0.6±0.2c
γ-Terpinene	0.5±0.1a	0.4±0.1a	0.4±0.2a	1.8±0.5a	2.0±0.4a	1.8±0.5a
**Oxygen-containing monoterpenes**						
Carvacrol	100.0±0.0a	100.0±0.0a	100.0±0.0a	100.0±0.0a	100.0±0.0a	100.0±0.0a
Citronellal	94.9±0.2a	47.5±0.1b	0.0±0.0d	1.1±0.3cd	2.1±0.4c	2.0±0.5c
Citral[1]	6.8±0.8a	3.5±0.4b	0.2±0.1c	1.8±0.5bc	2.2±0.4bc	1.8±0.5bc
Geraniol	100.0±0.0a	100.0±0.0a	100.0±0.0a	100.0±0.0a	100.0±0.0a	100.0±0.0a
Geranyl acetone	0.0±0.0c	0.6±0.1bc	1.1±0.3ab	1.3±0.3ab	2.1±0.2a	1.9±0.4ab
Linalool	0.9±0.2a	1.2±0.2a	1.5±0.4a	1.8±0.5a	2.0±0.4a	1.7±0.5a
Menthol	87.0±0.8a	88.7±0.8a	90.3±0.8a	57.7±1.4b	39.1±1.0c	6.9±0.4d
Pulegone	0.0±0.0b	0.0±0.0b	0.0±0.0b	0.5±0.2b	1.5±0.3a	0.4±0.2b
α-Terpineol	48.9±0.9c	62.3±0.6b	75.8±1.1a	31.3±0.6d	2.1±0.4e	1.8±0.5e
Terpinen-4-ol	14.8±0.8a	7.5±0.3b	0.3±0.2c	0.9±0.3c	1.8±0.4c	1.4±0.4c
**Sesquiterpene**						
trans-β-Caryophyllene	17.9±0.5a	9.1±0.3b	0.4±0.2c	0.7±0.2c	1.5±0.3c	0.8±0.3c
**Phenylpropanoids**						
trans-Anethole	0.0±0.0b	0.1±0.1b	0.2±0.0b	1.7±0.3a	2.0±0.2a	1.8±0.6a
Eugenol	29.9±0.9c	65.0±0.5b	100.0±0.0a	100.0±0.0a	100.0±0.0a	100.0±0.0a
**Salicylate**						
Methyl salicylate	92.2±0.3c	96.1±0.2b	100.0±0.0a	99.3±0.2a	97.0±0.5b	81.5±0.7d
**Methyl ketone**						
2-Undecanone	0.0±0.0b	0.1±0.1b	0.2±0.1b	1.8±0.5a	2.1±0.4a	1.7±0.3a
**Pesticide**						
Oxamyl[2]	73.9±1.3c	81.2±1.2b	83.3±1.4b	92.7±1.8a	95.3±1.2a	95.7±1.1a

[1] Citral is a mixture of the two geometric stereoisomers geranial (*trans*-citral) and neral (*cis*-citral), more commonly found in natural conditions. [2] The active compound of the pesticide Afromyl™.

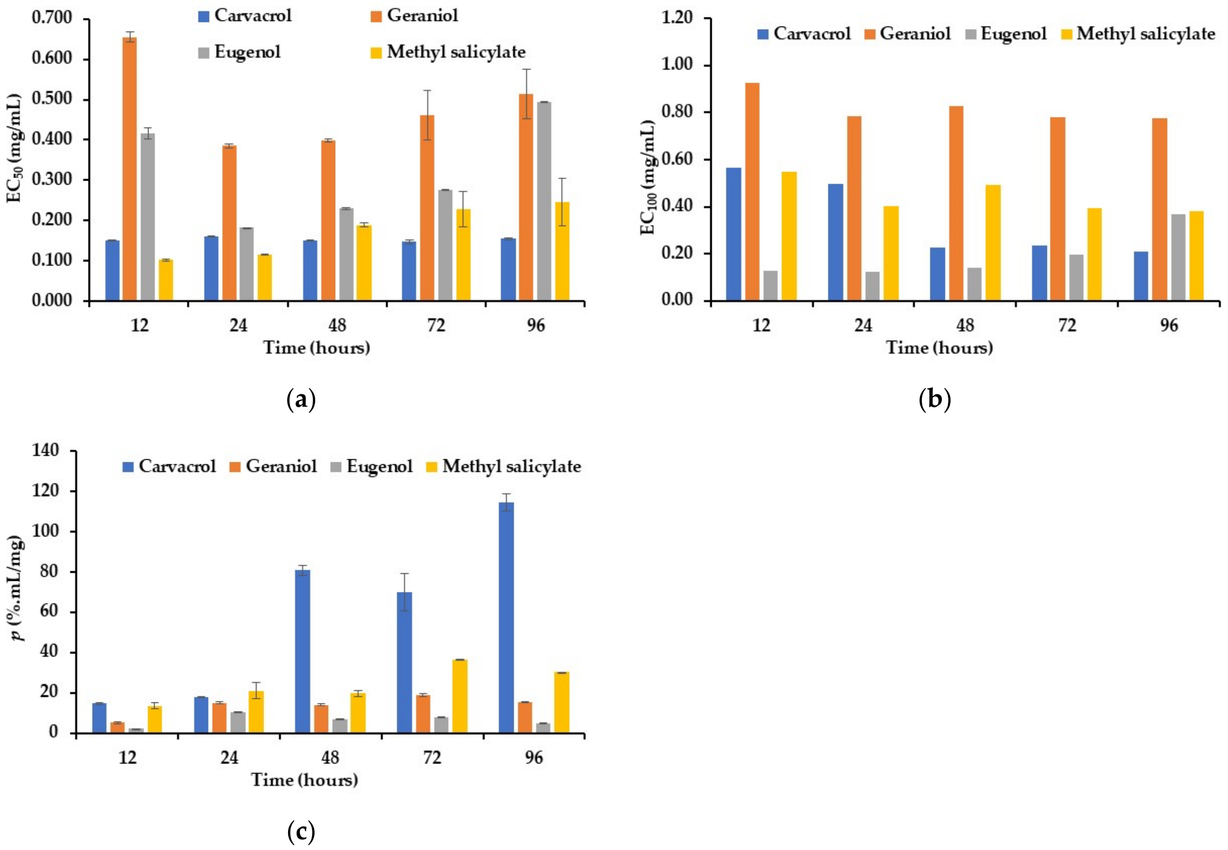

Supplementary Figure S1. Half-maximal effective concentration (EC_{50}, in mg/mL) (**a**) and lowest maximal effective concentration (EC_{100}, in mg/mL) (**b**) of carvacrol, eugenol, geraniol, and methyl salicylate on *M. graminicola*, obtained by fitting a dose-response sigmoidal curve. Slope (*p*) values are presented for comparison purposes (**c**).
